# Chloroflexota in agricultural soils: current knowledge and future research directions

**DOI:** 10.3389/fmicb.2026.1705889

**Published:** 2026-01-30

**Authors:** Jakub Dobrzyński, Marcin Gradowski, Adam Radkowski, Henryk Bujak

**Affiliations:** 1 Institute of Technology and Life Sciences – National Research Institute, Raszyn, Poland; 2 Department of Biochemistry and Microbiology, Institute of Biology, Warsaw University of Life Sciences, Warsaw, Poland; 3 Department of Agroecology and Plant Production, University of Agriculture in Krakow, Krakow, Poland; 4 Department of Genetics, Wroclaw University of Environmental and Life Sciences, Wroclaw, Poland; 5 Research Centre for Cultivar Testing, Slupia Wielka, Poland

**Keywords:** agricultural soils, life-history strategies, microbial ecology, soil bacterial predominants, soil indicators

## Abstract

The review organizes current knowledge on the biofunctions, life-history strategies, and environmental responses of Chloroflexota in agricultural soils. Members of this phylum play key roles in carbon, nitrogen, and phosphorus cycling through a high degree of metabolic versatility, including photosynthesis, redox reactions, and the degradation of complex organic compounds such as cellulose and lignin. Chloroflexota contribute to major soil processes, including nitrification, denitrification, and nitrogen fixation. In agricultural soils, the predominant classes are Anaerolineae and Ktedonobacteria, each exhibiting distinct ecological strategies. Anaerolineae members, such as *Leptolinea*, *Bellilinea*, and *Anaerolinea*, are often associated with nutrient-enriched conditions, suggesting copiotrophic or competitor- and ruderal-like traits. In contrast, Ktedonobacteria show negative responses to increased soil carbon and nitrogen, suggesting that its members are oligotrophic. Despite these trends, responses to soil organic carbon, nitrogen, phosphorus, and pH vary substantially across studies, likely due to functional heterogeneity within the phylum and insufficient taxonomic resolution in metataxonomic datasets. Emerging evidence from metagenome-assembled genomes (MAGs) reveals that Chloroflexota harbor genes involved in carbon fixation, nitrogen transformations, and phosphorus solubilization, highlighting their previously underestimated ecological significance. However, most Chloroflexota remain uncultured, and available genomic data are still limited. Future research integrating high-resolution taxonomic profiling, metagenomics, and cultivation-based approaches is needed to clarify the ecological roles and life-history strategies of Chloroflexota members. Such advances may ultimately establish this phylum as an important microbial indicator of soil fertility and environmental change in agricultural soils.

## Introduction

**Figure 1:**
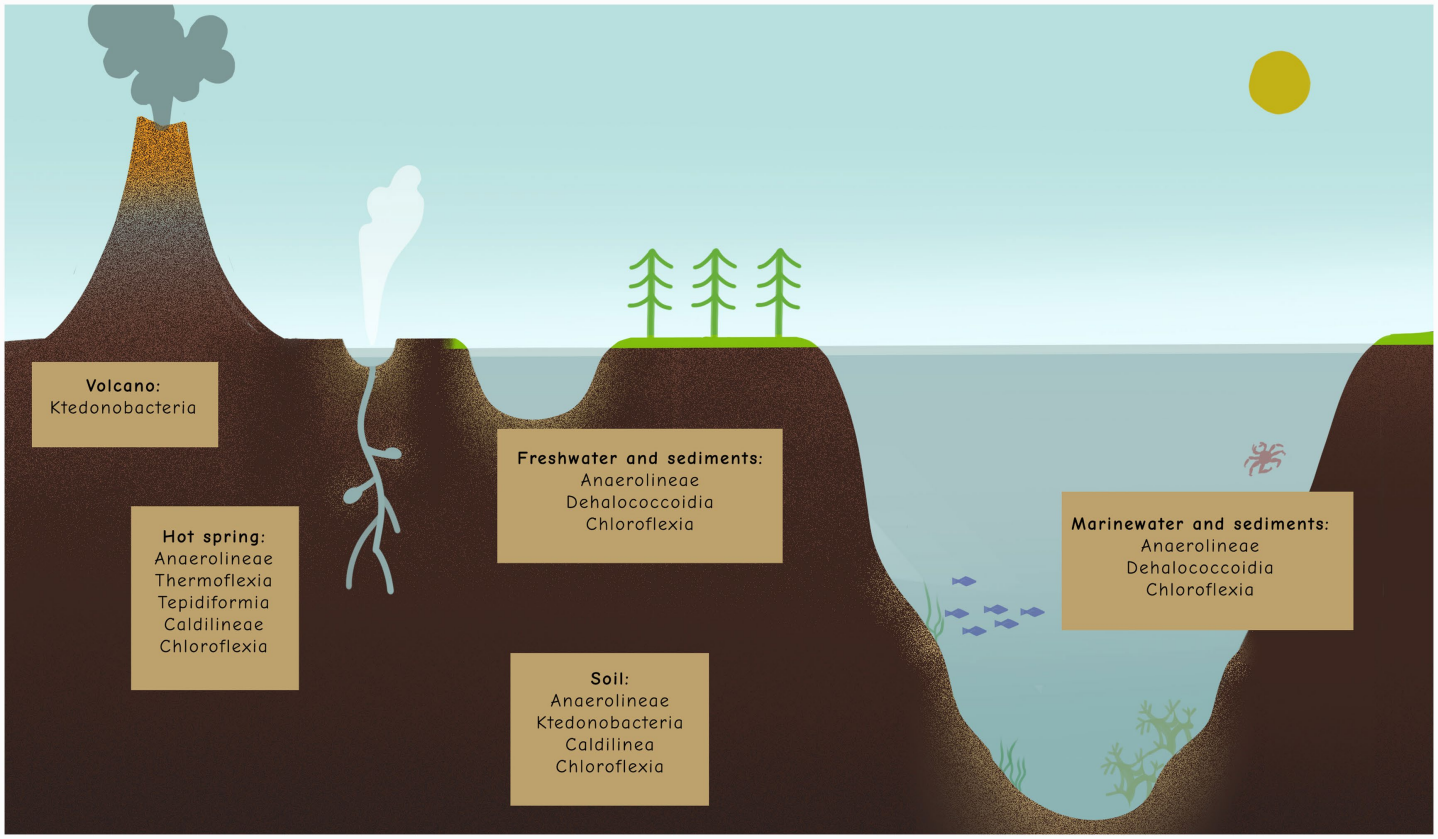
Distribution of Chloroflexota members (classes) in example environments.

The phylum Chloroflexota (formerly Chloroflexi), commonly referred to as green non-sulfur bacteria, represents a highly diverse group of bacteria inhabiting a wide range of environments. These include terrestrial ecosystems (e.g., soils, freshwater sediments, and hot springs) and aquatic systems (e.g., marine water and sediments) (Figure 1) (Ward et al., 2020; Wiegand et al., 2023). Additionally, Chloroflexota have been identified as part of the human microbiome, particularly in the gastrointestinal tract and oral cavity (Banar et al., 2024; Sánchez-Quinto et al., 2020).

The phylum derives its name from *Chloroflexus aurantiacus*, a filamentous anoxygenic phototroph first described by Pierson and Castenholz (1974). Over time, advances in phylogenetic analyses and the discovery of novel species have led to ongoing taxonomic revisions. Currently, Chloroflexota comprises several confirmed classes: Anaerolineae, Ardenticatenia, Caldilineae, Chloroflexia, Dehalococcoidia, Ktedonobacteria, Tepidiformia, and Thermoflexia. In addition, there are multiple proposed or candidate classes, including *Candidatus* Spiritibacteria*, Candidatus* Limnocylindria*, Candidatus* Martimicrobia*, Candidatus* Tarhunnaeia*, Candidatus* Uliximicrobia*, Candidatus* Bathosphaeria*, Candidatus* Poriflexia*, Candidatus* Thermomicrobiia*, and Candidatus* Umbricyclopia (Freches and Fradinho, 2024; Williams et al., 2024; Wang et al., 2025).

Chloroflexota members exhibit remarkable metabolic versatility, playing critical roles in biogeochemical cycles, particularly carbon (C) and nitrogen (N) transformations. Members of this phylum include cellulolytic bacteria (Hug et al., 2013; Zheng et al., 2021), nitrite oxidizers (Sorokin et al., 2012), and denitrifiers (Schwartz et al., 2022). While many studies have focused on Chloroflexota in marine environments (Wasmund et al., 2014; Hoshino et al., 2020) or wastewater treatment systems (Nierychlo et al., 2019), their relative abundance in soils—sometimes exceeding 20%—suggests a fundamental role in terrestrial ecosystems (Chen et al., 2017; Jia et al., 2020; Wu et al., 2022; Xu et al., 2019; Yang et al., 2019).

Current advances in sequencing technologies, particularly high-throughput Illumina platforms, enable tracking of microbial relative abundance through analysis of hypervariable regions (V1–V9) within the 16S rRNA gene (Regueira-Iglesias et al., 2023). Using this framework, Anaerolineae and Ktedonobacteria were identified as the predominant classes in soil-related communities within this phylum (Wan et al., 2021; Chen et al., 2017; Li et al., 2023; Qian et al., 2023), although other classes, such as Chloroflexia, Caldilineae, Thermomicrobia, Limnocylindria, and Dormibacteria, are also present (Ai et al., 2018; Chen et al., 2019; Cui et al., 2022; Wang et al., 2025).

Importantly, due to their relatively high abundance and metabolic diversity in soils, Chloroflexota are likely to play significant yet underexplored roles in soil ecosystem functioning. However, these roles have not been systematically reviewed, and existing knowledge gaps remain poorly characterized. A better understanding of the ecological functions and dynamics of Chloroflexota is essential for advancing insights into soil biogeochemical processes and for promoting sustainable soil ecosystem management (Ho et al., 2017).

Therefore, this review aims to synthesize current knowledge on the ecological functions of Chloroflexota in soil ecosystems and their responses to key soil chemical properties and fertilization practices. Specifically, it seeks to elucidate their functional roles in soil biochemical processes and life-history strategies in agricultural soils, while identifying gaps in their current understanding and suggesting directions for future research to address these gaps.

## Biofunctions and ecological roles of Chloroflexota

Members of the phylum Chloroflexota serve critical functions in various environments by participating in key biogeochemical processes. Their ability to perform photosynthesis under anaerobic conditions, participate in redox processes, and produce hydrolytic enzymes enables them to facilitate the mineralization of organic substances and the cycling of elements in nature (Freches and Fradinho, 2024).

### Carbon metabolism and ecological responses

**Figure 2:**
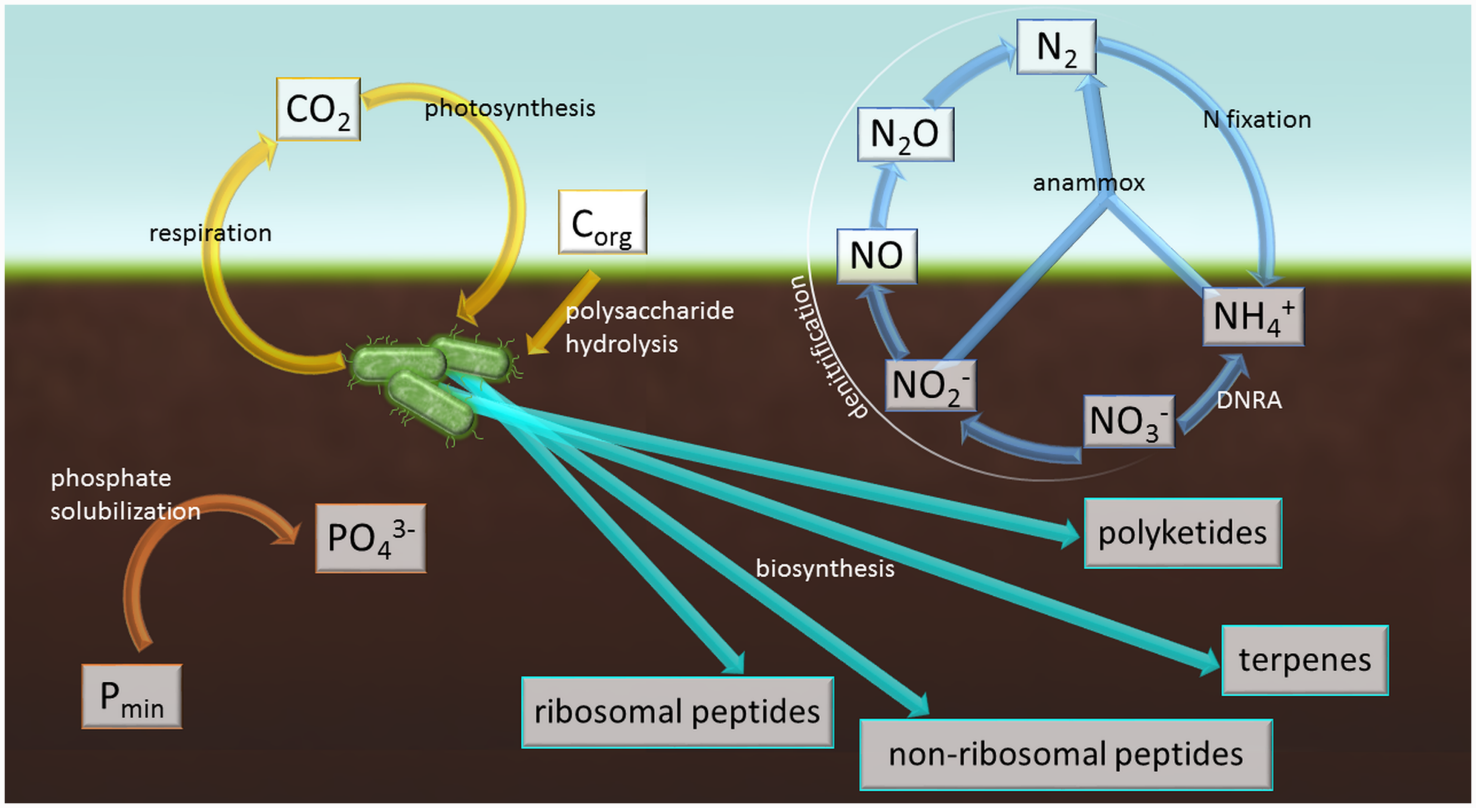
The role of Chloroflexota in element cycling and secondary metabolite biosynthesis in soil known to date.

The metabolic capabilities of Chloroflexota significantly influence the carbon cycle, particularly through photosynthesis and the breakdown of complex organic substances (Figure 2). Members of Chloroflexota are capable of producing key enzymes, such as exo- and endoglucanases and β-glucosidase, which are essential for cellulose degradation, and endo-beta-1,4-xylanases - involved in xylan hydrolysis (Xiong et al., 2023). For instance, members of the genera *Ornatilinea* (Anaerolineae class), *Ktedonobacter*, *Thermosporothrix*, and *Thermogemmatispora* (all three within the class Ktedonobacteria) exhibit the ability to degrade cellulose and starch (Podosokorskaya et al., 2013; Tomazini et al., 2018; Yan et al., 2020; Yang et al., 2019; Zheng et al., 2021). Additionally, members of the Dehalococcoidia class are known for their capacity to degrade lignin (Palmer et al., 2023). In bioreactors, members of Chloroflexota have demonstrated the ability to degrade organic matter, including xylan, indicating their potential role in the degradation of complex biopolymers (Bovio-Winkler et al., 2023). Moreover, their enzymatic abilities have been leveraged in biogas production within methanogenic bioreactors, such as those involving the *Bellilinea* or *Leptolinea* genera. Their enzymatic activity facilitates the fermentation and conversion of organic matter into methane, highlighting their importance in organic waste conversion processes (Bovio-Winkler et al., 2021).

Due to the pivotal role of Chloroflexota in the soil carbon cycle, their relative abundance often responds to the addition of carbon-containing organic amendments. However, studies have reported highly variable patterns of response (Iqbal et al., 2022; Kong et al., 2024; Rao et al., 2021), likely reflecting the broad diversity of life-history strategies (r- and K-strategists) and functional traits (e.g., distinct enzymatic profiles) among Chloroflexota taxa inhabiting soil ecosystems (Table 1).

**Table 1:** Correlation between soil carbon and the relative abundance of Chloroflexota.

Taxon level	Correlation (+/−)	Fertilization	Plants	Soil	Climate	Location	Sequencing platform/16S rRNA gene regions	References
Chloroflexota	–	Chemical fertilizer (NPK)	Long-Term Continuous Soybean Cropping System After Corn Insertion	Light chernozem (Chinese Soil Taxonomy -CST)	Cold temperate continental monsoon climate	Jilin Academy of Agricultural Sciences, China	Illumina MiSeq platform/V4	Rao et al. (2021)
Chloroflexota	–	Organic fertilizer, microbial fungal fertilizer, composite fertilizer,	Summer maize–winter wheat crop rotation system	Brown soil with sandy loam (CST)	Warm temperate, semi-humid, semi-arid monsoon climate	JXY Base of Beijing Miyun District, Chin	Illumina MiSeq platform/V3–V4	Kong et al. (2024)
Chloroflexota	–	Chemical (NPK and organic fertilizer)	Annual rotations of winter rape and summer rice	Haplic alisol (World Reference Base for Soil Resources—WRB)	–	Anji city, Zhejiang Province, China	Illumina HiSeq platform/V3–V4	Luo et al. (2023)
Chloroflexota	–	Chemical (NPK) and organic fertilizer	Rice-wheat rotation system	–	Humid subtropical monsoon climate	Changshu, Jiangsu province, China	Illumina MiSeq platform/V4	Wang Y. et al. (2016) and Wang J. et al. (2016)
Chloroflexota	–	Chemical fertilizer (NPK), organic fertilizer (cattle manure)	Rice and maize	Yellow paddy soil (CST)	Subtropical monsoon climate	Guizhou Academy of Agricultural Sciences, China	Illumina MiSeq platform/V4	Guo et al. (2019)
Chloroflexota	+	Urea and organic manure (cattle manure, poultry manure)	Rice	Ultisols (USDA Soil Taxonomy)	Sub-tropical monsoon climate	Rice research station of Guangxi University, Nanning, China	Illumina MiSeq platform/V3–V4	Iqbal et al. (2022)
Chloroflexota	+	Chemical fertilizer and biochar	Rice	–	temperate continental monsoon climate	Yesheng Town, Qingtongxia City, China	Illumina MiSeq platform/V3–V4	Sun J. et al. (2022)
Chloroflexota	+	Water-soluble humic acid fertilizer	Lei bamboo plantions	Red soil (CST)	Subtropical monsoon climate	Minjin, Deqing County, Huzhou City, China	Illumina NovaSeq platform/V3–V4	Ni et al. (2024)
Chloroflexota	–	Chemical fertilizer (NPK)	Chinese chives	Fluvisols (WRB)	Continental semi-arid climate.	Qingchi village, Wushan County, China	Illumina MiSeq platform/V3–V4	Niu et al. (2022)
Chloroflexota	–	Chemical fertilizer (NPK)	Rice	Typical sandy purple clay soil (CST)	Humid subtropical monsoon zone	Sanxianhu Village, Nan County, Yiyang City, Hunan Province, China	Illumina NovaSeq platform/V4	Xiao et al. (2023)
Chloroflexota	–	Chemical fertilizer (NPK)	Crop rotation of cucumber and tomato	Loamy clay soil (soil texture)	Plastic solar greenhouse	Shijiazhuang, Hebei Province, China	Illumina MiSeq platform//V3–V4	Sun N. et al. (2022)

For example, in a long-term NPK fertilization experiment involving soybean cultivation in China, a decline in Chloroflexota abundance was associated with higher soil organic carbon content (Rao et al., 2021). Similarly, a study utilizing organic and microbial fertilizers in a summer maize–winter wheat crop rotation system also reported reduced Chloroflexota abundance with increased soil organic carbon (Kong et al., 2024). Other studies reporting similar findings are summarized in Table 1. Conversely, some studies suggest a positive relationship between soil organic carbon (SOC) and Chloroflexota. For instance, Iqbal et al. (2022) documented an increase in Chloroflexota abundance in rice cultivation with higher SOC levels. As with the studies reporting negative correlations, these findings are also summarized in Table 1.

Considering the diverse responses of the entire Chloroflexota phylum to increased organic carbon content, further analysis at lower taxonomic levels is required. At this point, it should be noted that the dominant classes of Chloroflexota in agricultural soils are Anaerolineae and Ktedonobacteria. However, data on specific classes, orders, families, or genera within Chloroflexota remain limited. Jin et al. (2021) reported a significant positive correlation between the class Anaerolineae and total carbon (TC) in rice cultivation amended with biofertilizer. Biochar applications have also been shown to promote the growth of Anaerolineae in agricultural soils. Moreover, an increase in the Anaerolineae class following the application of organic fertilizers has also been observed at a lower taxonomic level. Gu et al. (2017) showed that manure rich in organic carbon, such as straw-based compost, has been linked to increased relative abundances of the genera *Leptolinea* and *Bellilinea* (class Anaerolineae). Additionally, manure fertilization was found to enhance the abundance of the genus *Thermomarinilinea* (class Anaerolineae) through positive correlations with organic matter (OM) (Lee et al., 2023). Similarly, in treatments with straw return, Yu et al. (2019) observed a significant increase in the relative abundance of the genus *Anaerolinea* (class Anaerolineae) in paddy field soil.

However, some studies, such as those on maize cultivation with long-term fertilization, reported no significant correlation between OM and the abundance of Anaerolineae (Sha et al., 2023). Other taxa, such as *Dehalogenimonas* (class Dehalococcoidia), exhibited increased abundance after the application of rice straw - a source of organic matter - but did not respond to inorganic fertilizers (Ahn et al., 2016). In summary, the above findings suggest that members of the class Anaerolineae, including the genera *Leptolinea* and *Belliline*a, may exhibit copiotrophic traits or be classified as competitors or ruderal under the Competitor–Stress-tolerator–Ruderal (C–S–R) framework (Ho et al., 2017); this topic is further addressed in the section “Life History Strategies of Chloroflexota Members.” Relating these patterns to the biochemical characteristics of Anaerolineae remains challenging, as the only well-characterized soil isolate to date is *Longilinea arvoryzae* KOME-1, obtained from paddy soil, which does not utilize xylose (Table 2) – a compound expected to increase in availability following manure application. Future research aimed at uncovering the functional diversity of these taxa is discussed in the section “Hidden Functions of Soil Chloroflexota.”

**Table 2:** Characterization of soil isolates of the phylum Chloroflexota.

Taxon	Class/family	General characteristics	Carbon transformation	Nitrogen transformation	Site of Isolation	References
*Ktedonobacter racemifer* SOSP1-21 T	Ktedonobacteria/Ktedonobacteraceae	Aerobic, mesophilic, filamentous, non-motile, spore-forming, Gram-positive, heterotrophic bacterium	Hydrolyzing starch, casein, gelatin, keratin but not cellulose, xylan	Not reduce nitrates	Soil sample of a black locust wood in Gerenzano, Northern Italy	Cavaletti et al. (2006), Chang et al. (2011)
*Ktedonosporobacter rubrisoli* SCAWS-G2T	Ktedonobacteria/Ktedonobacteraceae	Aerobic, mesophilic, filamentous, spore-forming, Gram-positive, heterotrophic bacterium	Utilize chitosan oligosaccharide, dextrin, d-galactose, but not hydrolase casein, cellulose, chitin, gelatin or starch	Reduced nitrates	red soil in Jiangxi Province, PR China	Yan et al. (2020)
*Dictyobacter aurantiacus* S-27 T	Ktedonobacteria/Dictyobacteraceae	Aerobic, mesophilic, Gram-stain-positive, spore-forming bacterium	Hydrolyse polysaccharides such as starch, cellulose (Avicel and xylan)	No data available	paddy soil in Gunung Salak (Mount Salak), West Java, Indonesia.	Yabe et al. (2017)
*Dictyobacter vulcani*	Ktedonobacteria/Dictyobacteraceae	Aerobic, mesophilic, Gram-stain-positive bacterium	Weakly hydrolase celullose and xylan and not hydrolase carboxymethylcellulose (CMC), but utilize D-glucose	No data available	soil of the Mt. Zao volcano in Miyagi, Japan	Zheng et al. (2020)
*Tengunoibacter tsumagoiensis* Uno3T	Ktedonobacteria/Dictyobacteraceae	Mesophilic, non-motile, Gram-positive, aerobic bacterium	Hydrylazing xylan but not CMC and celullose, utilize sucrose	No data available	soil-like granular mass, collected in an alpine area in Tsumagoi-mura, Gunma Prefecture, Japan.	Wang et al. (2019)
*Dictyobacter kobayashi* Uno11T	Ktedonobacteria/Dictyobacteraceae	Mesophilic, non-motile, Gram-positive, aerobic bacterium	Hydrolyzing xylan, celullose, and carboxymethylcellulose (CMC), and utilize sucrose and xylose	No data available	soil-like granular mass, collected in an alpine area in Tsumagoi-mura, Gunma Prefecture, Japan.	Wang et al. (2019)
*Longilinea arvoryzae* KOME-1	Anaerolineae/Anaerolineaceae	Obligately anaerobic, non-spore-forming, non-motile and Gram-negative bacterium	Utilize tryptone and pectin, not utilize starch and xylose	no data available	Rice paddy soil, Japan	Yamada et al. (2007)

### Nitrogen metabolism and ecological responses

Nitrogen is a crucial nutrient in agricultural soils; it directly influences the composition and activity of the soil microbial community, as many bacteria participate in nitrogen cycling processes (Geisseler et al., 2010) (Figure 2). Chloroflexota members also can play an important role in the nitrogen cycle. They participate in processes such as nitrification, denitrification, and atmospheric nitrogen fixation. However, only a few isolates capable of these activities have been identified (Lv et al., 2020; Bovio-Winkler et al., 2023), and even fewer are derived directly from soil (Table 2). For instance, the genus *Chloroflexales* (class Chloroflexia) includes members involved in anaerobic ammonia oxidation (anammox) (Lv et al., 2020). In the Chloroflexota including family Anaerolineaceae, metagenomic analyses have identified denitrification genes, including *nar* (nitrate reductase) and *nir* (nitrite reductase), as well as evidence of their ability to perform DNRA (dissimilatory nitrate reduction to ammonium) (Keren et al., 2020; Lawson et al., 2017; Bovio-Winkler et al., 2023). Furthermore, *Ardenticatena maritima* (the class Ardenticatenia) has been shown to carry out denitrification via a pathway involving nitrate reductase, nitrite reductase, nitric oxide reductase, and nitrous oxide reductase (Hemp et al., 2015). Nitrogen fixation through nitrogenase activity has been detected, inter alia, in members such as *Dehalococcoides mccartyi* (class Dehalococcoidia) (Koirala and Brözel, 2021). Beside, the strain *Oscillochloris trichoides* DG6 (class Chloroflexia) possesses a set of *nif* genes, including *nifH*, *nifD*, *nifK*, and *nifB*, which also indicates a genetic potential for atmospheric nitrogen fixation (Ivanovsky et al., 2021).

The role of Chloroflexota members in nitrogen turnover suggests that the composition of their community may be sensitive to variations in nitrogen availability resulting from agricultural fertilization practices. Since members of this phylum participate in multiple nitrogen transformation processes, including nitrogen fixation, mineralization, nitrification, and denitrification, changes in external nitrogen inputs can markedly influence both their abundance and functional activity. Fertilization with nitrogen-rich compounds may selectively promote certain nitrogen-transforming bacterial groups (Aasfar et al., 2021; Vijayan et al., 2021) while simultaneously reducing overall diversity, potentially leading to shifts in soil ecosystem balance (Beltran-Garcia et al., 2021; De Souza and Freitas, 2018; Sabir et al., 2021). These effects are likely to vary among Chloroflexota members, depending on their specific nitrogen utilization strategies and ecological niches; for example, different types of fertilizers may favor distinct physiological groups within the phylum. Nitrogen occurs in agricultural soils in various forms, but its largest inputs are via fertilization, including chemical fertilizers such as ammonium nitrate or urea, and organic fertilizers such as manure or slurry (Grzyb et al., 2021). As with other bacteria, urea application may enhance the growth of ureolytic and ammonifying populations (Sun et al., 2019b; Abdo et al., 2022), whereas diazotrophic populations may decline under high mineral nitrogen availability, as external inputs suppress biological nitrogen fixation (Berthrong et al., 2014; Yin et al., 2015). However, in the case of Chloroflexota, current knowledge remains too limited to draw definitive conclusions, and these statements should be considered hypotheses requiring experimental validation.

Several studies report a negative correlation between total nitrogen (TN) and the abundance of Chloroflexota (Niu et al., 2022; Rao et al., 2021; Sun N. et al., 2022). For instance, Rao et al. (2021) observed such a pattern in a study of fertilization (NPK) in continuous soybean and soybean-maize rotation. Other studies do not directly correlate TN with Chloroflexota but indicate that nitrogen fertilization - whether inorganic or organic - decreases the relative abundance of this phylum. For example, Ullah et al. (2020) demonstrated a negative effect of mineral nitrogen fertilization (N) on the relative abundance of Chloroflexota in a winter wheat and summer maize crop rotation system. These patterns have been observed in numerous other studies focusing on nitrogen fertilization (Eo and Park, 2016; Ai et al., 2018; Sun et al., 2019a; Turlapati et al., 2013; Wang et al., 2023; Yao et al., 2014; Zhou et al., 2017). Additionally, there are reports of negative correlations between ammonium or nitrate nitrogen and Chloroflexota (Ren et al., 2024; Xu et al., 2022).

Conversely, some studies show a positive correlation between TN and Chloroflexota. For example, Iqbal et al. (2022) observed such a pattern in rice cultivation under organic and chemical fertilization. Similarly, Gu et al. (2017) detected a positive correlation between Chloroflexota and TN in a comparable experiment. Other studies have reported similar findings (Ahn et al., 2016; Liu et al., 2023; Morigasaki et al., 2024; Niu et al., 2022; Sun J. et al., 2022). Positive correlations between Chloroflexota and nitrate nitrogen have also been reported by Feng et al. (2024) and Ren et al. (2020). Moreover, studies have noted a positive effect of nitrogen fertilization on Chloroflexota without establishing direct correlations with TN (Wang Y. et al., 2016; Liu et al., 2017; Cui et al., 2018; Guo et al., 2019; Guo et al., 2020; Wang et al., 2017; Yuan et al., 2016; Zhu et al., 2022).

In general, as previously mentioned, the response of the entire Chloroflexota phylum to nitrogen or nitrogen fertilization appears inconsistent, which, as mentioned above, is probably due to considerable heterogeneity among its members in terms of soil nitrogen metabolism. Similar to the case of carbon, insights should be sought at lower taxonomic levels.

For the predominant class Anaerolineae, the response to nitrogen also varies. Tong et al. (2023) observed an increase in the relative abundance of Anaerolineae following urea fertilization in an alfalfa monocropping and maize rotation experiment. The class Anaerolineae also responded positively to biofertilizer and organic fertilizer applications in rice cultivation; the fertilization contained relatively high amounts of nitrogen (Jin et al., 2021). In a study on rice and wheat cultivation, (Wang Y. et al., 2016; Wang J. et al., 2016) found a noticeable increase in Anaerolineae in soil fertilized with N (urea) PK compared to the control. Positive effects of nitrogen fertilization on Anaerolineae have been noted in other studies using manure and NPK fertilization (Yi et al., 2018; Zarraonaindia et al., 2020; Li et al., 2022). Organic and NPK fertilization also increased the relative abundance of the order Anaerolineales (the class Anaerolineae) in paddy field studies (Chen et al., 2017). Similar relationships have also been observed at lower taxonomic levels. For instance, Gu et al. (2017) observed significantly higher abundances of *Leptolinea* and *Bellilinea* in soil amended with a mixed fertilizer (chemical fertilizer and farmyard manure) that increased ammonium nitrogen compared to the control. However, some studies have reported opposite trends. For example, Yao et al. (2020) found a negative impact of urea fertilization on the relative abundance of Anaerolineae. Similar trends have been observed in other studies, where the family Anaerolineaceae showed reduced relative abundance following urea fertilization (Qian et al., 2023; Zhao et al., 2022). Bacteria of the class Caldilinea have also shown varying responses to nitrogen (Chen et al., 2019; Cui et al., 2022).

Taken together, these studies indicate that members of the class Anaerolineae, including the family Anaerolineaceae, are associated both with urea-fertilized soils – the most commonly applied nitrogen fertilizer – and with unfertilized soils, highlighting their context-dependent and variable responses to nitrogen inputs. However, these observations are largely based on high-level taxonomic assignments, and additional data at lower taxonomic levels (e.g., genus or species) are needed to disentangle the ecological drivers behind these contrasting patterns. Some insight could be gained from the review of the currently available Anaerolineae isolates; yet, as noted above, only a single soil isolate is known – *Longilinea arvoryzae* KOME-1—which has not been characterized for nitrogen compound transformations. Consequently, formulating hypotheses regarding the response of Anaerolineae to nitrogen fertilization remains challenging.

For the class Ktedonobacteria, including the order Ktedonobacterales, responses to increased nitrogen content in the soil appear more consistent. Most studies indicate a negative response of Ktedonobacteria to nitrogen fertilization. Wan et al. (2021) reported a negative correlation between urea fertilization and the relative abundance of Ktedonobacteria in a long-term nitrogen fertilization experiment in citrus orchard soils. Li et al. (2023) observed a higher relative abundance of Ktedonobacteria in unfertilized soil when biofertilizer (*Aspergillus brunneoviolaceus* HZ23) increased TN content. Zhang et al. (2022) also associated the order Ktedonobacterales with unfertilized soil in a study where inorganic (NPK) and organic fertilization increased ammonium and nitrate nitrogen. Similar patterns were reported in a study by the research group of Chen et al. (2017). In addition, there are a number of studies showing the effect of NPK on Chloroflexota abundance without determining a correlation between this phylum and nutrients (Table 3). Collectively, these observations suggest that the negative response of members of Ktedonobacteria to increased soil nitrogen availability may reflect their limited involvement in nitrogen cycling processes. However, this interpretation is not fully supported by the available biochemical data, which moreover are derived from only a limited number of soil isolates. For example, *Ktedonobacter racemifer* SOSP1-21 T does not reduce nitrates, whereas *Ktedonosporobacter rubrisoli* SCAWS-G2T is capable of nitrate reduction; yet such information is available for only a very limited number of strains (Table 2). For most described soil isolates within Ktedonobacteria, including members of the genera Dictyobacter and Tengunoibacter, data on nitrogen transformation are lacking. Consequently, the small number of well-characterized soil isolates and the scarcity of information on their nitrogen metabolism make it difficult to draw definitive conclusions regarding the role of Ktedonobacteria in soil nitrogen cycling.

**Table 3:** Influence of NPK fertilization on Chloroflexota (without determining the correlation with individual nutrients).

Fertilization	Influence on relative abundance of Chloflexota	Plants	Soil	Climate	Location	Sequecing platform/16S rRNA gene regions	References
NPK and biochar (alone and both)	Increased	One annual crop of peanuts	Hapli-Udic Cambisol (WRB)	Semi-humid climate	Liaoning Province, China, at the Shenyang Agricultural University Peanut Scientific Research Center	Illumina MiSeq platform/V4–V5	Gao et al. (2021)
NPK	No reaction	Paddy field soil (long-term experiment)	Fine silty mixed mesic Typic Haplaquepts (USDA)	Mild temperate climate	Department of Functional Cereal Crop Research Farm, Miryang, South Korea	454 GS FLX—pyrosequencing/no data	Daquiado et al. (2016)
NPK	Decreased	Double-cropping system with wheat and corn	Cambisol (WRB)	Temperate continental monsoon climate	Luancheng Agro-Ecosystem Experimental Station, Chinese Academy of Sciences	Illumina MiSeq platform	Liu et al. (2020)
NPK	No reaction	Wheat-soybean crop rotation	Black soil (CST)	Humid subtropical climate	Mengcheng county, Anhui province	454 GS FLX—pyrosequencing/V4–V5	Sun et al. (2015)
NPK	Increased	Tea (*Camellia sinensis*) plantation	Red soil (CST)	Humid subtropical climate	5 large tea plantations located in different regions of Hunan province	Illumina MiSeq platform/V4	Gu et al. (2019)
NPK	No reaction	Maize	Inceptisol (USDA)	Warm temperate climate	National Field Science Research Stations of the Chinese Academy of Sciences [29]: Hailun station in Heilongjiang Province of northern China	No data available	Ni et al. (2021)
NPK	Increased	Century-long continuous winter wheat	-	Humid subtropical climate	Central Oklahoma, USA	454 FLX/FLX + pyrosequencing/ V1–V9	Li et al. (2020)
NPK	Decreased	Leguminosae, Cruciferae and Tuber crop rotation pattern.	Quaternary red clay soil (Q), granite soil (G) and purple sandy shale (P) (CST)	Subtropical monsoon climate	Qiyang, Hunan province, China	454 GS FLX—pyrosequencing/V1–V3	Sun et al. (2016)
NPK	Increase in the class Anaerolineae	Long-term chili (Capsicum spp. L.)	Yellow brown soil (CST) (alfisol)	Subtropical monsoon climate	Yuhang County, in northern Zhejiang Province, China	Illumina MiSeq platform/V3–V4	Yi et al. (2018)

Alternatively, metataxonomic analyses suggest that the observed decline in Ktedonobacteria under elevated nitrogen availability may be linked to their life-history strategies rather than a direct role in nitrogen transformations. The negative correlation with nitrogen availability may indicate that Ktedonobacteria are predominantly oligotrophic or stress-tolerant taxa within the C–S–R framework. In this context, changes in nutrient availability may indirectly affect Ktedonobacteria by altering ecological niches and resource competition, as discussed in the following section (Life strategies of Chloroflexota members).

### Phosphorus and potassium effects on soil Chloroflexota

Phosphorus and potassium are essential macronutrients that are of fundamental importance to the functioning of soils and their microbiome. Members of Chloroflexota can participate in phosphorus transformations, although most studied strains derive from water and sediments (Freches and Fradinho, 2024; Madueño et al., 2021; Miura et al., 2007). Nevertheless, in soils, genes such as *phoD* (encoding alkaline phosphatase) and other genes involved in enhancing soil phosphorus availability have been detected using PCR and high-throughput sequencing (Ragot et al., 2015; Wang et al., 2025) (Figure 2). In the study by Wang et al. (2025), Chloroflexota MAGs (Metagenome-Assembled Genomes) harbored diverse phosphorus solubilization genes, including (i) *gcd* (encoding quinoprotein glucose dehydrogenase)—primarily affiliated with the classes Chloroflexia, Limnocylindria, and UBA5177; (ii) *phnP* (encoding C-P lyase subunit)—mainly affiliated with the classes Anaerolineae and Limnocylindria; (iii), *phoA* (alkaline phosphatase) and *phoD*, primarily affiliated with the classes Ktedonobacteria, Limnocylindria, and Anaerolineae. Many MAGs also contained *pepM*, a gene responsible for phosphonate production (mainly affiliated with Ktedonobacteria and UBA5177), which may enhance microbial P storage in P-limited environments. Additionally, the classes Limnocylindria and Dehalococcoidia were identified as valuable taxa for P solubilization, as many of their MAGs carried multiple P solubilization genes (Wang et al., 2025). Collectively, the presence of these genes suggests that Chloroflexota may contribute to the transformation of phosphorus supplied in fertilizers, both organic (e.g., compost, manure) and mineral (e.g., rock phosphate).

Likely due to the heterogeneity of phosphorus transformation capabilities within Chloroflexota, the relationship between soil phosphorus content and their relative abundance varies across studies. However, the majority of reports indicate a negative correlation between available phosphorus and Chloroflexota abundance. For instance, fertilization with phosphorus-based fertilizers significantly reduced the relative abundance of Chloroflexota in paddy soils (Liu et al., 2020). Similar patterns were observed in paddy fields by Samaddar et al. (2019) and Yu et al. (2019). A negative correlation between Chloroflexota and available phosphorus (AP) was also reported in Chinese chive cultivation under different fertilization treatments (Niu et al., 2022). Other researchers have also documented such relationships (Sun N. et al., 2022; Wu et al., 2022; Zhang et al., 2022). According to our knowledge, fewer studies have reported a positive effect of phosphorus on the relative abundance of Chloroflexota. For instance, Tong et al. (2023) observed a positive correlation between available phosphorus (AP) and Chloroflexota in alfalfa monocropping fertilized with nitrogen. Notably, Gu et al. (2017) reported a beneficial effect of phosphorus on this bacterial phylum, in contrast to the findings of Liu et al. (2020), Samaddar et al. (2019), and Yu et al. (2019). Similar correlations have also been documented in other studies (Eo and Park, 2016; Liu et al., 2023).

These results highlight the apparent metabolic diversity within Chloroflexota, which is inferred largely from the varied responses of its members rather than from confirmed isolate-based or genomic evidence (e.g., MAGs). This suggests that the effects of phosphorus fertilization on Chloroflexota should be examined at lower taxonomic levels. However, the literature on this topic remains limited. For example, Lee et al. (2023) reported a positive correlation between available phosphorus and bacteria from the genus *Thermomarinilinea* in soil fertilized with Korean manure. Similarly, isolate-based evidence is scarce – not only due to the difficulty of cultivating Chloroflexota, but also because studies reporting isolates seldom examine genes or enzymes involved in phosphorus cycling (Cavaletti et al., 2006; Yabe et al., 2017; Yan et al., 2020).

In the case of potassium, no precise information is currently available on which Chloroflexota genes may be involved in its cycling. Nevertheless, most studies indicate a negative correlation between soil potassium content and the relative abundance of the Chloroflexota. For instance, an analysis involving several cropping systems, including continuous maize and continuous sweet potato cultivation, demonstrated that potassium content negatively correlates with the relative abundance of Chloroflexota (Alami et al., 2021). Similar trends have been observed in Chinese chive cultivation under different fertilization regimes (Niu et al., 2022). Additional studies have reported comparable correlations (Kong et al., 2024; Sun et al., 2016; Wu et al., 2020; Yang et al., 2024b). Nevertheless, some studies have reported contrary findings, indicating a positive correlation between potassium and Chloroflexota (Liu et al., 2023; Yan et al., 2021). Similar to phosphorus, research on the effects of potassium at lower taxonomic levels within Chloroflexota remains scarce, and the existing data are inconclusive. For example, no correlation has been observed between potassium content and the family Anaerolineaceae during long-term fertilization of a corn field in Shanghai (Sha et al., 2023).

### Impact of soil pH on the Chloroflexota

The influence of pH on soil microbiota, including bacterial communities, is a crucial factor in shaping the structure and functioning of soil ecosystems. Soil pH impacts the availability of nutrients and heavy metals, which in turn determine the composition of microbiota (Xiong et al., 2024). In agricultural soils, changes in soil pH – such as those induced by liming or acidification – can result in significant shifts in structures and functions of microbial communities, directly affecting soil productivity and ecosystem health (Geisseler and Scow, 2014; Wang et al., 2019). Generally, acidic soils favor the dominance of taxa such as the phylum Acidobacteriota, whose representatives are mostly well-adapted to low pH conditions and nutrient-poor environments (Wang et al., 2019; Wierzchowski et al., 2021; Górska et al., 2024). Conversely, bacteria within the phyla Actinobacteriota, Proteobacteria, and Bacteroidota tend to prefer neutral to alkaline soils. Similarly, the phylum Firmicutes is more prevalent in soils with higher pH and richer organic matter content (Barka et al., 2016; Kruczyńska et al., 2023; Spain et al., 2009; Wang et al., 2019).

The response of the Chloroflexota phylum to pH changes is highly variable, likely due to the broad metabolic diversity among its lower taxonomic ranks (e.g., family and genera). This functional heterogeneity explains why pH fluctuations can either increase or decrease the relative abundance of Chloroflexota in different soil environments. For example, in long-term fertilization experiments involving N, P, and manure in winter wheat cultivation, a significant negative correlation was observed between pH and the relative abundance of Chloroflexota (Wang Y. et al., 2016; Wang J. et al., 2016). Moreover, lower pH levels appeared to favor the development of Chloroflexota in teak plantations (Zhang et al., 2022) and other experiments which is presented in Table 4 (Alami et al., 2021; Jin et al., 2021; Niu et al., 2022; Sun J. et al., 2022; Yan et al., 2021; Zhang et al., 2017).

**Table 4:** Correlation between pH and the relative abundance of Chloroflexota.

Taxon level	Correlation (+/−)	Fertilization	Plants	Soil (classification)	Climate	Location	Sequecing platform/ 16S rRNA gene regions	References
Chloroflexota	–	Chemical fertilizer (no detailed data)	Eight different continuous cropping fields (and 1 fallow field)	Soil types mostly sandy and clay (soil texture)	Humid subtropical climate	Jiannan county, Lichuan City, Hubei province, China	Illumina MiSeq platform/ V4–V5	Alami et al. (2021)
Chloroflexota	–	Organic, NPK and CaMgP fertilizers	Teak Plantations	Yellow−red earth (CST)	Humid subtropical monsoon climate	Luodian county, Qiannan Prefecture, Guizhou Province, China	Illumina Novaseq platform/ V3–V4	Zhang et al. (2022)
Chloroflexota and Anaerolineae (class)	–	Biofertilizer, organic and NPK fertilizers	Rice cultivation	SX soil was characterized as silt clay loam and TZ soil was silt loam (soil texture)	Greenhouse conditions (pot experiment in Zhejiang University)	The soil originated from Shaoxing (SX) city and Taizhou (TZ) city in Zhejiang province, China	Ion Torrent platform/ V4–V5	Jin et al. (2021)
Chloroflexota	–	NPK fertilizer	Chinese chive cultivation	Fluvisols (WRB)	Humid subtropical climate	Qingchi village, Wushan County, China	Illumina MiSeq platform/ V3–V4	Niu et al. (2022)
Chloroflexota	–	Biochar and N amendment	Rice cultivation	Soil bulk density was 1.56 g cm − 3 (no specific data)	Humid subtropical monsoon climate	Yesheng Town, Qingtongxia City, China	Illumina MiSeq platform/ V3–V4	Sun J. et al. (2022)
Chloroflexota	+	NPK fertilizer	Winter wheat-summer maize rotation	Ferric Acriso (WRB)	Humid subtropical monsoon climate	Jingdong county, Yunnan Province, China	PacBio Sequel platform/ V1–V9	Zhao et al. (2022)
Chloroflexota	+	NPK fertilizer	GrassMan experiment	Haplic Cambisol (WRB)	Temperate oceanic climate	Solling Uplands, Germany	454 GS FLX - pyrosequencing/ V2–V3	Herzog et al. (2015)
Chloroflexota	+	NPK fertilizer	Cucumber-tomato rotation	Loamy clay soil (soil texture)	Temperate continental monsoon climate	Shijiazhuang, Hebei Province, China	Illumina MiSeq platform/ V3–V4	Sun N. et al. (2022)
Chloroflexota	+	NPK and boric fertilizers	Rice-oilseed rape rotation	Yellow soil (CST)	Humid subtropical monsoon climate	Heishi Village Qionglai City, Sichuan Province	Illumina MiSeq platform/ V4	Xu et al. (2019)
Chloroflexota	+	NPK and organic fertilizers	Wheat cultivation	No data available	Temperate oceanic climate	Rothamsted Research, Harpenden, United Kingdom	Illumina MiSeq platform// V4	Kavamura et al. (2018)
SHA-31 family (Anerolineae class)	+	NPK fertilizer	Tomato and Swiss chard plants	No data available	Temperate oceanic climate	Basque Country, Spain	Illumina MiSeq platform/ V4	Zarraonaindia et al. (2020)
Anaerolineaceae	+	NPK and NPK and manure fertilizers	wheat–rice rotation	Anthrosol, paddy soil (WRB, CST)	Humid subtropical monsoon climate	Dujiangyan Sichuan Province, Southwest China.	Illumina MiSeq platform/ V1–V9	Li et al. (2020)

Conversely, a comparable number of metataxonomic studies reported a positive relationship between increasing pH and the relative abundance of Chloroflexota. For instance, a study on long-term fertilization in subtropical southwestern China found a positive correlation between pH levels and the relative abundance of this phylum (Zhao et al., 2022). Similar observations were made by Herzog et al. (2015) in a fertilization experiment conducted on grassland soils and in studies on long-term fertilized rice fields (Ahn et al., 2016). Additional studies supporting this trend include those by Kavamura et al. (2018), Sun N. et al. (2022), Xu et al. (2014, 2019), among others.

At lower taxonomic levels, data remain sparse, making it difficult to establish patterns. Nevertheless, Jin et al. (2021) observed a negative effect of increasing pH on the relative abundance of the Anaerolineae class in rice fields. In contrast, Zarraonaindia et al. (2020) reported a positive correlation between soil pH and the relative abundance of the SHA-31 family, belonging to the class Anerolineae. Similarly, Li et al. (2022) documented a positive correlation between the family Anaerolineaceae and pH in a wheat–rice rotation system (Table 4).

### Hidden functions of soil Chloroflexota

Despite their ecological importance – sometimes accounting for up to 20% of the soil microbial community – most Chloroflexota species remain uncultivated, genomically uncharacterized, and represented by only a few isolated strains (Freches and Fradinho, 2024). Examples of soil isolates (and from soil-like granular mass) and their characteristics are presented in Table 2. As noted in the previous section, the limited availability of data and the insufficient understanding of the functional roles of these taxa in soil make it difficult to explain the observed changes in their abundance in response to factors such as fertilization practices or soil organic carbon content.

While traditional cultivation approaches have been hindered by the fastidious growth requirements and symbiotic dependencies of many Chloroflexota members (Matsuura et al., 2015; Jia et al., 2016), recent advances in sequencing technologies and bioinformatics offer promising solutions.

The rapid development of high-throughput sequencing, particularly long-read platforms (PacBio, Nanopore), together with improved bioinformatic tools, is revolutionizing our ability to reconstruct MAGs (metagenome-assembled genomes) and assign functional genes to specific taxa. These advances are expected to enable more accurate taxonomic classification of Chloroflexota sequences from complex environmental samples, including soil.

Despite these opportunities, MAG-based knowledge of Chloroflexota remains limited. However, recently, Wang et al. (2025) reconstructed 170 medium- to high-quality Chloroflexota MAGs from agricultural soils (paddy soil, maize soil, and tea soil) co-contaminated with arsenic (As) and antimony (Sb) in Guizhou Province (Qinglong and Dushan counties), China. Among these, 11 MAGs were proposed as novel candidate species, including 3 novel candidate genera belonging to the classes Ktedonobacteria, Limnocylindria, and Dormibacteria. Functional annotation revealed that many Ktedonobacteria and Dormibacteria MAGs may possess novel carbon fixation potential via the Calvin–Benson–Bassham (CBB) cycle, and thatnumerous Chloroflexota MAGs carried key genes involved in enhancing soil phosphorus (P) availability (Wang et al., 2025).

Similarly, Zhang et al. (2024) analyzed agricultural soil microbiomes across all 31 Chinese provinces using metagenomics and identified 8,303 gene cluster families (GCFs) in MAGs. Their study revealed that Chloroflexota encode diverse secondary metabolites, including nonribosomal peptide synthetases (NRPS), terpenes, and polyketide synthases (PKS) (Figure 2). Notably, *RippS* (ribosomally synthesized and post-translationally modified peptide) genes were specifically linked to Chloroflexia class, highlighting previously unrecognized biosynthetic potential within this lineage.

Despite these advances, genome-resolved approaches face challenges related to differential sequence conservation across genetic markers. Certain housekeeping genes involved in core metabolism can vary significantly even between strains of the same species, enabling accurate taxonomic resolution, whereas functional genes involved in nitrogen cycling, such as *nifA–nifL* (nitrogen fixation) or *nirK/nirS* (denitrification), are often highly conserved even across distantly related taxa (Zhang et al., 2023; Yang et al., 2024a, 2024b). This conservation can result in ambiguous taxonomic assignments when relying solely on metagenomic or metatranscriptomic data.

While emerging technologies will undoubtedly advance ecological understanding of Chloroflexota, genomic predictions must ultimately be validated through complementary novel approaches. The integration of improved sequencing technologies, advanced bioinformatics, and refined cultivation strategies will be crucial for disentangling the true diversity and ecological significance of this abundant yet understudied phylum in soil ecosystems. Importantly, omics-based research already suggests potential functions related to plant growth promotion, and in combination with novel cultivation methods, Chloroflexota may eventually serve as biocontrol agents or direct growth promoters, contributing to the development of sustainable agriculture (Jakubowska et al., 2025; Naziębło and Dobrzyński, 2025; Dobrzyński and Kulkova, 2025).

### Life history-strategies of Chloroflexota members

The classical r/K life-history framework distinguishes organisms based on their reproductive and growth strategies and provides a useful lens for interpreting the ecological behavior of Chloroflexota in soil. In this model, r-strategists are typically copiotrophic organisms that thrive under nutrient-rich conditions by displaying rapid growth and resource exploitation. In contrast, K-strategists are considered oligotrophic, adapted to resource-limited but stable environments, and capable of maintaining slow yet efficient nutrient utilization (Ho et al., 2017).

Early studies predominantly classified Chloroflexota as oligotrophs (Davis et al., 2011; Ling et al., 2017; Pepe-Ranney et al., 2016), largely based on their occurrence in low-nutrient habitats and slow growth rates. However, this view appears increasingly incomplete in light of recent findings. Several studies have reported positive correlations between relative abundance of Chloroflexota and elevated soil organic carbon or nitrogen, suggesting that some members may exhibit copiotrophic traits (Niu et al., 2022; Iqbal et al., 2022; Sun J. et al., 2022; Ni et al., 2024). Collectively, these observations indicate that the phylum likely encompasses both r- and K-strategists, depending on the taxonomic level and environmental context, consistent with its broad metabolic diversity and the diverse ecological strategies exhibited by its constituent lineages.

At lower taxonomic levels, available metataxonomic data suggest that Anaerolineae and Ktedonobacteria are the dominant Chloroflexota classes in agricultural soils (Chen et al., 2017; Gu et al., 2017; Ai et al., 2018; Wang et al., 2020; Zhao et al., 2022; Amadou et al., 2020). Within Anaerolineae, genera such as *Anaerolinea*, *Leptolinea*, and *Bellilinea* (Anaerolineaceae family) are frequently associated with nutrient-enriched or manure-amended soils, indicating potential copiotrophic behavior (Gu et al., 2017; Jin et al., 2021; Lee et al., 2023; Yu et al., 2019). Conversely, several studies have reported negative correlations between Anaerolineae abundance and nitrogen fertilization, although the taxonomic resolution in these datasets does not allow identification of whether *Anaerolinea*, *Leptolinea*, or *Bellilinea* were specifically affected (Qian et al., 2023; Zhao et al., 2022; Yao et al., 2020). In contrast, members of Ktedonobacteria appear to favor nutrient-poor or unfertilized soils, suggesting a more oligotrophic lifestyle (Zhang et al., 2022; Li et al., 2023; Huo et al., 2024). Similarly, taxa such as *Roseiflexus* (class Chloroflexia) have been reported to increase in non-fertilized soils, further supporting this interpretation (Ai et al., 2018).

Given this complexity, the Competitor–Stress-tolerator–Ruderal (C–S–R) framework (Ho et al., 2017) may provide a more nuanced conceptual model than the classical r/K dichotomy. Under this framework, Anaerolineae associated with high organic carbon or nitrogen may function as Competitors (C), characterized by efficient utilization of available nutrients that supports rapid growth. In contrast, Ktedonobacteria and Chloroflexia members inhabiting nutrient-limited soils could represent Stress-tolerators (S), capable of maintaining activity under resource scarcity. Transient increases in organic inputs, such as manure or straw application, may temporarily favor Ruderals (R), likely among fast-responding Anaerolineae taxa.

## Conclusions, limitations, and future perspectives

The phylum Chloroflexota comprises a phylogenetically and physiologically diverse group of soil bacteria. Although many species remain uncultured due to persistent cultivation challenges (Freches and Fradinho, 2024), recent advances in metagenome-assembled genomes (MAGs), high-throughput sequencing, and bioinformatics have been significantly improving our understanding of their functional roles in carbon and nitrogen cycling, phosphorus availability, and secondary metabolite production (Wang et al., 2025; Zhang et al., 2024).

Chloroflexota responses to soil properties – such as pH, organic carbon, nitrogen, phosphorus, and potassium – are highly variable. For example, Anaerolineae frequently respond positively to nutrient-enriched soils, whereas Ktedonobacteria tend to favor more oligotrophic conditions (Gu et al., 2017; Jin et al., 2021; Zhang et al., 2022). This heterogeneity underscores the need for analyses at finer taxonomic levels to accurately interpret ecological patterns and assess life-history strategies of the taxa (Chen et al., 2017; Ai et al., 2018). A major limitation of current research is the insufficient genus- and species-level resolution in both metataxonomic and MAG-based analyses, which hinders comprehensive ecological interpretations. Future research should therefore emphasize large-scale meta-analyses using 16S rRNA sequencing or assembled genome data to better characterize species-level diversity and metabolic potential (Freches and Fradinho, 2024; Wang et al., 2025).

Finally, experimental studies of Chloroflexota response to different fertilization strategies – including PGPB applications – combined with MAG-based analyses and continued cultivation advances, could reveal key ecological functions and identify lower-level taxa as potential bioindicators of soil quality (Dobrzyński et al., 2024, 2025a, 2025b).
